# Transplantation of mesenchymal stromal cell-derived mitochondria alleviates endothelial dysfunction in pre-clinical models of acute respiratory distress syndrome

**DOI:** 10.1093/stcltm/szaf053

**Published:** 2025-11-15

**Authors:** Dayene de Assis Fernandes Caldeira, Johnatas Dutra Silva, Monique Martins Melo, Rodrigo Gonzaga Veras, Daniel F McAuley, Patricia Rieken Macedo Rocco, Pedro Leme Silva, Fernanda Ferreira Cruz, Anna Krasnodembskaya

**Affiliations:** Wellcome-Wolfson Institute for Experimental Medicine, School of Medicine, Dentistry, and Biomedical Sciences, Queen’s University Belfast, Belfast BT9 7BL, United Kingdom; Laboratory of Pulmonary Investigation, Institute of Biophysics Carlos Chagas Filho, Federal University of Rio de Janeiro, Rio de Janeiro CEP: 21941-902, Brazil; Wellcome-Wolfson Institute for Experimental Medicine, School of Medicine, Dentistry, and Biomedical Sciences, Queen’s University Belfast, Belfast BT9 7BL, United Kingdom; Laboratory of Pulmonary Investigation, Institute of Biophysics Carlos Chagas Filho, Federal University of Rio de Janeiro, Rio de Janeiro CEP: 21941-902, Brazil; Laboratory of Pulmonary Investigation, Institute of Biophysics Carlos Chagas Filho, Federal University of Rio de Janeiro, Rio de Janeiro CEP: 21941-902, Brazil; National Institute of Science and Technology for Regenerative Medicine, Rio de Janeiro, Rio de Janeiro, Brazil; Laboratory of Pulmonary Investigation, Institute of Biophysics Carlos Chagas Filho, Federal University of Rio de Janeiro, Rio de Janeiro CEP: 21941-902, Brazil; National Institute of Science and Technology for Regenerative Medicine, Rio de Janeiro, Rio de Janeiro, Brazil; Wellcome-Wolfson Institute for Experimental Medicine, School of Medicine, Dentistry, and Biomedical Sciences, Queen’s University Belfast, Belfast BT9 7BL, United Kingdom; Laboratory of Pulmonary Investigation, Institute of Biophysics Carlos Chagas Filho, Federal University of Rio de Janeiro, Rio de Janeiro CEP: 21941-902, Brazil; National Institute of Science and Technology for Regenerative Medicine, Rio de Janeiro, Rio de Janeiro, Brazil; Laboratory of Pulmonary Investigation, Institute of Biophysics Carlos Chagas Filho, Federal University of Rio de Janeiro, Rio de Janeiro CEP: 21941-902, Brazil; National Institute of Science and Technology for Regenerative Medicine, Rio de Janeiro, Rio de Janeiro, Brazil; Laboratory of Pulmonary Investigation, Institute of Biophysics Carlos Chagas Filho, Federal University of Rio de Janeiro, Rio de Janeiro CEP: 21941-902, Brazil; National Institute of Science and Technology for Regenerative Medicine, Rio de Janeiro, Rio de Janeiro, Brazil; Wellcome-Wolfson Institute for Experimental Medicine, School of Medicine, Dentistry, and Biomedical Sciences, Queen’s University Belfast, Belfast BT9 7BL, United Kingdom

**Keywords:** acute respiratory distress syndrome, mesenchymal stromal cells, mitochondrial dysfunction, mitochondrial transplantation, pulmonary endothelial barrier

## Abstract

**Background:**

Pulmonary endothelial dysfunction with increased capillary permeability is a key aspect in the pathogenesis of acute respiratory distress syndrome (ARDS). It has been demonstrated that mesenchymal stromal cells (MSC) can modulate host cells through mitochondrial transfer. Although mitochondrial transplantation is a promising treatment strategy for conditions underpinned by mitochondrial dysfunction, its therapeutic potential in ARDS has not been sufficiently investigated. Herein, we tested the potential of MSC mitochondrial transplantation to restore functionality of the pulmonary endothelium in pre-clinical models of ARDS.

**Methods:**

Mitochondria (mt) derived from human bone-marrow MSC were isolated and immediately used for transplantation to primary human pulmonary microvascular endothelial cells (HPMEC) in the presence of *Escherichia coli* lipopolysaccharide (LPS) or plasma samples from ARDS patients classified into hypo- and hyper-inflammatory phenotypes. Mitochondrial function, inflammatory status, and barrier integrity of HPMEC were assessed at 24 h. LPS- challenged mice were treated with MSC-mt intravenously, and the severity of lung injury and inflammatory response were evaluated.

**Results:**

Exposure to LPS or ARDS plasma induced endothelial hyperpermeability associated with mitochondrial dysfunction. MSC-mt were readily internalized by HPMEC without cytotoxicity or inflammatory response, mitigating mitochondrial dysfunction and restoring barrier integrity. In vivo, administration of MSC-mt alleviated lung injury, reduced inflammatory cell infiltration in the alveoli and increased VE-cadherin mRNA levels in the lung tissue, indicating restoration of the alveolar-capillary barrier integrity.

**Conclusion:**

This study demonstrated MSC mitochondrial transplantation as a promising therapeutic approach for treatment of endothelial dysfunction in the context of acute inflammation. Further exploration of mitochondrial transplantation in ARDS is warranted.

## Graphical abstract

**Graphical Abstract:**
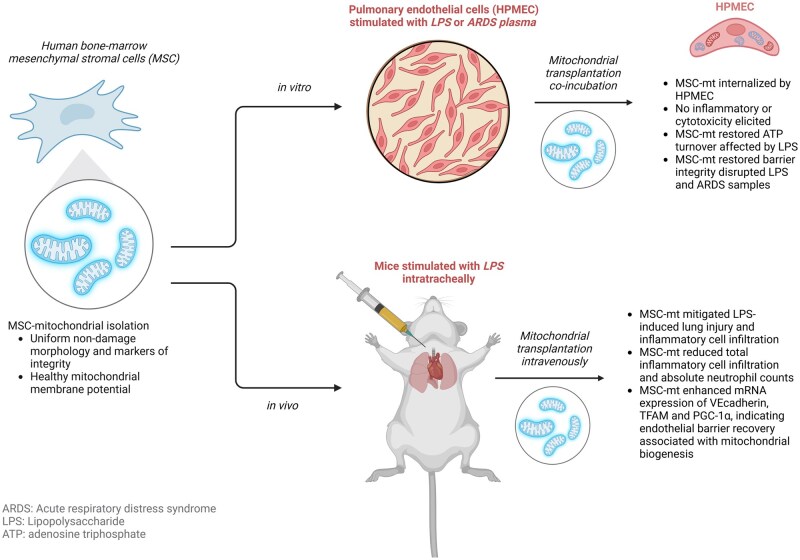

Significance Statement Mitochondrial dysfunction in pulmonary endothelial cells plays critical role in development of vascular leakage during acute respiratory distress syndrome (ARDS). Here we demonstrated that transplantation of mitochondria isolated from mesenchymal stromal cell (MSC) has therapeutic potential to restore endothelial barrier integrity disrupted by inflammatory stimuli. Our findings reveal that MSC-derived mitochondria can effectively mitigate capillary hyperpermeability and inflammation without cytotoxic effects in both *in vitro* and *in vivo* models. This innovative approach may pave the way for new treatments targeting endothelial dysfunction in ARDS, ultimately improving patient outcomes in this life-threatening condition.

## Introduction

Acute respiratory distress syndrome (ARDS) is characterized by dysregulated lung inflammation associated with impaired alveolar epithelial–endothelial barriers.^1^^,^^2^ This disorder culminates in fluid accumulation in the distal airspaces (pulmonary edema) and the acute onset of dyspnea, hypoxemia, and systemic inflammation.^1–3^ Several etiologies can trigger ARDS, from primary respiratory infections to trauma, and frequently it is associated with sepsis and multi-organ dysfunction ­syndrome.^4^ Two biological phenotypes of ARDS, hypo- and hyperinflammatory, have been classified according to plasma levels of inflammatory biomarkers, epithelial and endothelial injury, and coagulation abnormalities, and remarkably, these subphenotypes also have different clinical outcomes and differentially respond to treatment.^5^

In ARDS, pulmonary endothelial cells acquire a pro-inflammatory phenotype, with increased permeability, resulting in vascular leakage.^6^ Notably, hyper-permeability in pulmonary endothelial cells also involves mitochondrial depolarization in vivo.^7^ Previously, we have demonstrated that exposure of primary human pulmonary microvascular endothelial cells (HPMEC) and ex vivo cultured precision human lung slices (PCLSs) to plasma of ARDS patients induced mitochondrial dysfunction. We also showed that mitochondrial respiration was downregulated after LPS injury in the murine lung tissue in vivo.^8^ Observational studies suggest that mitochondrial dysfunction correlates with higher mortality in critically ill septic patients,^9^ while in survivors of multiple organ dysfunction syndrome (MODS), mitochondrial function was better maintained with preservation of ATP levels and mitochondrial biogenesis pathway.^10^

Mesenchymal stromal cells (MSC) and their secretome have been extensively studied for their repair ability and therapeutic potential.^11^ In particular, intercellular mitochondrial transfer between MSC and damaged tissue via tunnelling nanotubules or extracellular vesicles was shown as an efficient physiological mechanism to replace dysfunctional mitochondria in experimental models of acute lung injury,^12^ asthma,^13^ chronic obstructive pulmonary disease (COPD),^14^^,^^15^ ARDS,^8^^,^^16^ fibrosis,^17^ and sepsis.^18^ Artificial mitochondrial transplantation is increasingly recognized as a promising method for treating conditions underpinned by mitochondrial dysfunction. In the present work, we tested the therapeutic potential of transplantation of mitochondria isolated from MSC in the restoration of the functionality of pulmonary endothelium in pre-clinical in vitro and in vivo models of ARDS.

Some findings of this study have been previously reported in abstract form.^19^^,^^20^

## Methods

### Cell culture

Human Bone-Marrow Mesenchymal Stem/Stromal Cells (MSC) were obtained from the American Type Culture Collection (ATCC PCS-500-012) and cultured according to the instructions provided. These cells fulfil all criteria established by the International Society of Cellular Therapy for defining MSC.^21^ MSC were cultivated in a T175 flask (ThermoFisher Scientific, UK) with 15 mL alpha-Minimum Essential Medium (α-MEM) supplemented with 16.5% heat-inactivated fetal calf serum (FCS), 1% L-glutamine amino acid (4 mM), and 1% penicillin/streptomycin (50 µg/mL) (all from Gibco, ThermoFisher Scientific, UK) and maintained at 37 °C in a humidified tissue culture incubator at 5% CO_2_ until used for experimentation. Culture medium was refreshed every 2–3 days, and cells were subcultured into new flasks whenever 70%–80% confluency was achieved. Upon reaching 80% confluence, media was aspirated from the flask of cells, washed with phosphate-buffered saline (PBS) to remove any FCS-containing medium, and MSC were passaged with 0.05% trypsin solution (Gibco, ThermoFisher Scientific, UK) for up to 5 min at 37 °C until the cells had detached from the culture surface. Trypsin activity was neutralized by the addition of α-MEM complete medium at a volume equal to that of the trypsin added to the flask, and cells were collected and centrifuged for 5 min at 300×*g*. Following centrifugation, the supernatant was aspirated off and the cell pellet was resuspended in α-MEM complete medium. Cell viability, density, and final concentration were then determined by Trypan Blue exclusion and by counting in a hemocytometer (Neubauer model). MSC were used in passages 4–6.

Primary human pulmonary microvascular endothelial cells (HPMEC) (Innoprot, Spain) were cultured in accordance with the manufacturer’s instructions and as described previously. Briefly, cells were cultured in T75 flasks (ThermoFisher Scientific, UK) in a humidified tissue culture incubator at 37 °C with 5% CO_2_ until used for experimentation. Cell passages were done when 80% confluency was reached.

Human monocytic cell line THP-1 (TIB-202, ATCC, UK) were maintained in suspension at 2 × 10^5^ to 8 × 10^5^ cells/mL in RPMI-1640 supplemented with 10% serum heat-inactivated fetal bovine (ThermoFisher Scientific, UK) and 1% penicillin/streptomycin (Sigma-Aldrich). For experiments, THP-1 monocytes were plated (2 × 10^4^ cells in a 96-well plate) and differentiated into macrophage-like cells with a working solution of 100 nM phorbol 12-myristate-13-acetate (PMA, Sigma-Aldrich) in supplemented medium for 72 h. The differentiated cells were left in a PMA-free medium for 24 h and the experiments were conducted afterward. The supernatants used were centrifuged at 180×*g* for 10 min and stored at −20 °C until analysis.

### Mitochondria isolation and characterization

Mitochondria Isolation Kit for Cultured Cells (ThermoFisher Scientific, UK) was used to isolate mitochondria, following the manufacturer’s instructions based on density centrifugation.^22^ Mitochondria preparations were made with 5 × 10^5^ MSC. Mitochondria were maintained on ice and used immediately for transplantation. For flow cytometry and confocal microscopy characterization, MSCs were pre-stained with 200 nM of Mitotracker Deep Red FM in 10 mL serum-free MEM in the dark for 45 min. Cells were washed with PBS, and the mitochondria were isolated. The final mitochondrial pellet was washed twice and resuspended in PBS. Gating of MSC mitochondria (MSC-mt) was performed based on FSC/SSC parameters. Analysis was conducted on a FACS CantoII flow cytometer using FACSDiva software, and data were analyzed with FlowJo software (FlowJo, Ashland, OR). Distinct samples of pre-stained and isolated mitochondria were visualized on a STELLARIS confocal microscope (Leica Microsystems, ­Danaher Corp., Germany). In addition, the presence of cytochrome C in samples of isolated mitochondria was analyzed by Western blot.

### Mitochondria transplantation in in vitro models of ARDS

MSC and HPMEC mitochondria were pre-labelled with fluorescent mitochondrial probes Mitotracker (ThermoFisher Scientific, UK) as described above. Next, HPMEC (2 × 10^4^ cells/well) were exposed to LPS (*Escherichia coli*, 0111: B4, Merck) at a concentration of 1 µg/mL of basal medium to mimic inflammatory microenvironment.^8^ In distinct experiments, plasma samples from ARDS patients recruited to the HARP-2 clinical trial and previously classified into either hypo- and hyper-inflammatory subphenotypes based on the levels of inflammatory biomarkers were used for stimulation. Baseline samples from 10 patients (5 from each phenotype) were pooled and then diluted to a final concentration of 10% before stimulation. Plasma samples from three healthy volunteers were used as controls. The Office for Research Ethics Committees, Northern Ireland, approved the research involving patient samples. Immediately after the exposure to LPS/ARDS plasma samples, HPMEC were co-incubated with 5 μg/well of MSC mitochondria (based on Mt protein concentration) for 24 h.

### Determination of mitochondrial DNA copy numbers and protein quantification

mtDNA copy numbers in MSC mitochondria (MSC-mt) preparation were evaluated as a surrogate for mitochondrial mass. Briefly, DNA was extracted from samples using a commercially available kit (DNeasy, Qiagen). mtDNA was measured using SYBR green I (Roche, Basel, Switzerland) by real-time-quantitative polymerase chain reaction (RT-qPCR, Life Technologies, Carlsbad, CA) for the mitochondrial *ND1* gene, measured in triplicate. The ND1 was used for mtDNA quantification, as it demonstrated excellent correlation with other mitochondrial genes used for mtDNA quantification.^24^ The primer sequences are as follows: MTND1 (forward, 5′-ATACCCATGGCCAACCTCCT-3′ and reverse 5′-GGGCCTTTGCGTAGTTGTAT-3′). The copy number was log-transformed per microliter to ensure normality and was shown in anti-log form. Protein concentrations were measured using BCA protein assay (Micro BCA protein assay kit, ThermoFisher Scientific, UK).

### Animal experiments

This study received approval from the Ethics Committee of the Health Sciences Center (CEUA-004/20) at the Federal University of Rio de Janeiro. All animals were maintained following the guidelines of the National Society for Medical Research and by the U.S. National Academy of Sciences. This study adheres to the ARRIVE guidelines for reporting animal research. Animals were housed under controlled temperature (23 °C) and light–dark cycle (12–12 h), with unrestricted access to water and food.

C57BL/6 male mice (8–12 weeks old, 20 ± 2 g) were randomly allocated to groups and used to induce lung injury. Investigators remained blinded to group allocation throughout the study and were only unblinded after the completion of data analysis. Mice were anaesthetized by inhalation of isoflurane 2.0% (Isoforine; Cristália, Brazil) and LPS (2 mg/kg of body weight) or vehicle (PBS) was instilled intratracheally.^8^ After 4 h, MSC mitochondria (5 µg/g of body weight) or vehicle (buffer) were given as treatment via jugular vein. On the following day (24 h after treatment), after euthanasia, lungs and bronchoalveolar lavage fluid (BALF) were collected for analysis.

### Real-time polymerase chain reaction

Lung tissue was immediately snap-frozen in liquid nitrogen and stored at −80 °C. Total RNA was extracted using RNeasy extraction kit (Promega, USA). From total RNA, cDNA was synthesized using the QuantiTect Reverse Transcription kit (Qiagen). mRNA levels were quantified using SYBR green-based RT-qPCR (QuantStudio, 5 Real-Time PCR System, Thermo Fisher Scientific). Gene expression in each sample was normalized to the housekeeping gene (acidic ribosomal phosphoprotein P0, 36B4) and expressed relative to control group. Analyzes were carried out using the 2^-ΔΔCT^ method. The following genes were evaluated: *TFAM* F: GAGGCAAAGGATGATTCGGCTC, R: CGAATCCTATCATCTTTAGCAAGC; *PGC1α* F: CGGAAATCATATCCAACCAG, R: TGAGGACCGCTAGCAAGTTTG; *VE-CAD* F: GAACGAGGACAGCAACTTCACC, R: GTTAGCGTGCTGGTTCCAGTCA; *36B4* F: CAACCCAGCTCTGGAGAAAC, R: GTTCTGAGCTGGCACAGTGA.

### Assessment of mitochondrial membrane potential

JC-1 (5,5ʹ, 6,6ʹ-tetrachloro-1,1ʹ, 3,3ʹ-tetraethylbenzimidazolylcarbocyanine iodide) dye was used to assess mitochondrial membrane potential. This dye accumulates within mitochondria and is dependent on mitochondrial potential. Red/green JC-1 fluorescence ratio is proportional to mitochondrial membrane potential. HPMEC were seeded at a density of 2 × 10^4^ in a 96-well black plate with a clear bottom in triplicate and incubated in standard cell culture conditions. Cells were stimulated with 1 µg/mL LPS (*E. coli*, 0111: B4, Merck) to generate an inflammatory microenvironment and treated with isolated mitochondria (5 µg/well). For this analysis, only unlabeled MSC-mt were used to avoid any possible bias due to the MitoTracker staining. After 24 h of stimulation, cells were stained with JC-1 (Abcam, 20 µM) for 15 min at 37° C in the dark. As a positive control, a pretreatment with carbonyl cyanide-p-trifluoromethoxyphenylhydrazone (FCCP, 100 µM) was used before JC-1 staining to induce mitochondrial depolarization. Wells were washed using a 1× dilution buffer. Live cells were promptly imaged at 20× magnification, using the EVOS FL Auto epifluorescent microscope. Fluorescence intensities for red and green channels were measured with ImageJ, and the red/green ratio was determined.

### Detection of mitochondrial reactive oxygen species (MitoSOX)

MitoSOX Red (ThermoFisher Scientific, UK), a fluorescent probe that specifically targets mitochondrial superoxide in live cells, was used to measure mitochondrial superoxide production. HPMEC were seeded at a density of 2 × 10^4^ in a 96-well black plate with a clear bottom (Nunc, ThermoFisher Scientific, UK) in triplicate and incubated in normal cell culture conditions. As a negative control, cells pretreated with 5 μM of MitoTempo (MT), a mitochondria-targeted superoxide dismutase antioxidant, 4 h prior to stimulation, were included. Next, cells were stimulated with 1 µg/mL LPS (*E. coli*, 0111: B4, Merck) and treated with MSC-mt. Following 24 h of cell stimulation, supernatants were collected and stored. MitoSOX (5 μM) was added to the cell culture and incubated for 20 min at 37 °C in the dark. After the staining, cells were washed and counterstained with 20 μM Hoechst (ThermoFisher Scientific, UK; 10 μM), and immediately imaged at 40× magnification using the EVOS FL Auto epifluorescent microscope. Red fluorescence intensity was quantified using ImageJ.

### Confocal imaging

To follow the internalization of mitochondria, some fluorescence analysis of the assays/experiments was done using the high-resolution confocal system Leica SP8 (Leica Microsystems, Germany). Imaging on the live cells was done in cultured cells in DMEM (without phenol red)/FCS 5%, with a 40× immersion objective. Tridimensional reconstructions were done using the iMARIS software.

### Enzyme-linked immunosorbent assay

Human IL-8 and TNF-α levels were measured using ELISA Duoset kits (R&D System, Biotechne) following the manufacturer’s instructions. Plates were read using VersaMax spectrophotometer (450 and 540 nm). Absorbance readings at 540 nm were subtracted from those at 450 nm for correction. Four-parameter standard curves were generated using Softmax Pro v2.6, and sample concentrations were determined by extrapolation.

### Determination of cell cytotoxicity by lactate dehydrogenase

LDH levels were measured using the Cytotoxicity Detection Kit (Roche) according to the manufacturer’s instructions. Cell supernatants were centrifuged at 250×*g* for 10 min at 4 °C to remove debris. Subsequently, 50 µL samples were added in triplicate into a 96-well plate (Nunc, ThermoFisher Scientific, UK). The reaction mixture was added to the samples, mixed on a plate shaker for 1 min, and incubated for 30 min in the dark at RT. As a positive control, cells were lysed with 2% TritonX (Sigma-Aldrich) 10 min before the collection of supernatants. Optical densities were measured at 405 nm using a FLUOstar Omega microplate reader. Data was analyzed using MARS data analysis software. Results are presented as percent relative to the positive control.

### Mitochondria respiration measurements

To determine the cells’ oxygen consumption rate (OCR), Agilent Seahorse XF Analyzers (Seahorse Bioscience) were used as previously published.^8^ Briefly, HPMEC (2 × 10^4^ cells/well) were stimulated with LPS (*E. coli*, 0111: B4, Merck) at a concentration of 1 µg/mL and treated with vehicle or MSC-mt for 24 h. Mitochondrial stress test was performed using XF assay medium (10 mM glucose, 1 mM pyruvate, and 2 mM glutamine, all from Agilent) and the following inhibitor concentrations: 1.5 μM oligomycin, 2.0 μM carbonyl cyanide p-trifluoromethoxyphenylhydrazone (FCCP), and 0.5 μM ­rotenone/antimycin-A (Rot/AA) (all from Agilent). After the addition of each inhibitor to the well, three sequential measurements were made. Normalization was done based on the percentage of OCR. Parameters for the mitochondrial stress were calculated using the Wave software (Version 2.2) (Agilent Technologies) following the manufacturer’s instructions.

### Cell impedance measurements

The xCELLigence RTCA SP system (Agilent) was used to measure, process, and analyze the impedance (cell index) detected in an E-plate 16 (ACEA Biosciences). Briefly, after HPMEC were seeded and formed an adherent monolayer, cells were stimulated with LPS or ARDS plasma samples. Immediately after, cells were treated with MSC-mt or vehicle for 24 h. The results were normalized based on cell index values before the addition of stimulations. Supernatant was collected and stored for further analysis.

### Statistical analysis

Analyzes were performed using the software GraphPad Prism 8 (GraphPad Software, La Jolla, CA, USA). Experiments were done at least in triplicate, and the average was calculated and taken as a single data point, later grouped for statistical analysis. For parametric data, Student’s *t*-test or one-way ANOVA with post hoc analysis using Tukey’s test were used. For non-parametric data, the Mann–Whitney *U* test or Kruskal–Wallis with post-hoc analysis using Dunn’s test was used. Data were presented as median ± standard deviation (SD) or median and interquartile range as appropriate. Significance was considered when *P* < .05.

## Results

### Mitochondria characterization

**Figure 1.**
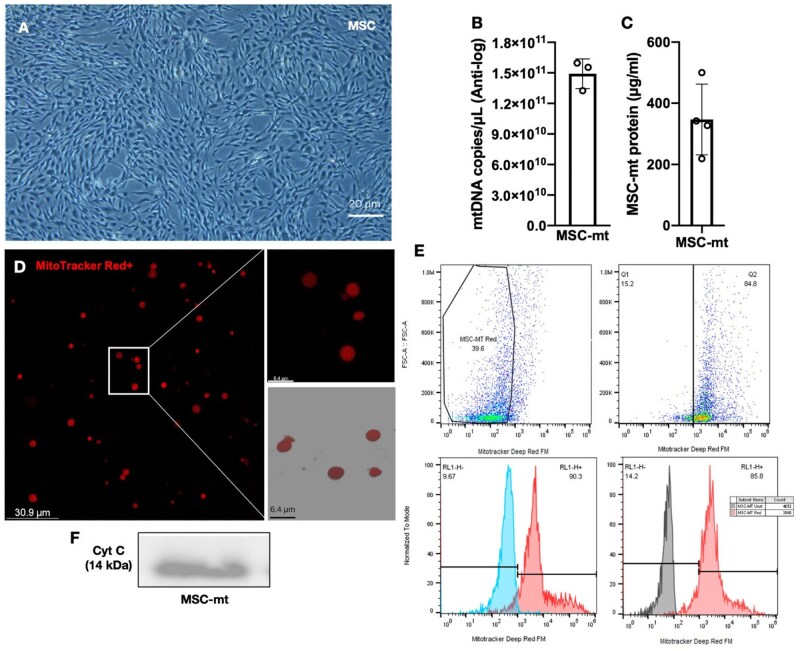
Characterization of MSC isolated mitochondria. (A) MSC in 2D culture before mitochondrial isolation. (B) Quantification of mtDNA copy number in MSC-mitochondria samples as a surrogate of mitochondrial mass. (C) Quantification of protein concentration in MSC-mt lysates. (D) Representative live images of respiration competent MitoTracker Red positive labelled MSC-mt. (E) Representative flow cytometry gate and histogram plots of MSC-mt prestained with MitoTracker Deep Red. MSC-mt samples had 87.5% ± 3.9% of particles positive for MitoTracker Deep Red FM (histograms of two independent measurements). (F) Western blot of Cytochrome C in MSC-mt lysates. Data presented as mean ± SD.

MSC were cultured in T175 flasks until 80%–90% confluence immediately before mitochondrial isolation (Figure 1A). Mitochondria were isolated from 5 × 10^5^ cells from three different MSC donors. The mtDNA copy number of MSC-mt preparations was 1.5 × 10^11^ ± 1.5 × 10^10^ copies/µL and the total protein content was 347.0 ± 115.9 μg/mL (Figure 1B and C). To evaluate the purity and fitness of isolated MSC-mt, before isolation, MSC were pre-stained with Mitotracker Deep Red dye, which only accumulates in actively respiring mitochondria. By confocal microscopy, MSC-mt preparation displayed uniform non-damaged round morphology (Figure 1D). By flow cytometry, a mean of 87.5% ± 3.9% of the mt preparation was positive for Mitotracker Deep Red indicating potential contamination with other organelles and/or non-functional mitochondria lower than 11.9% ± 3.0% (two independent measurements) (Figure 1E). Western blotting of the mitochondrial pellet demonstrated expression of cytochrome c (a marker of the inner membrane structure) (Figure 1F). Together, these data suggest the integrity of isolated mitochondria preparations.

### MSC-mt were efficiently internalized by HPMEC

**Figure 2.**
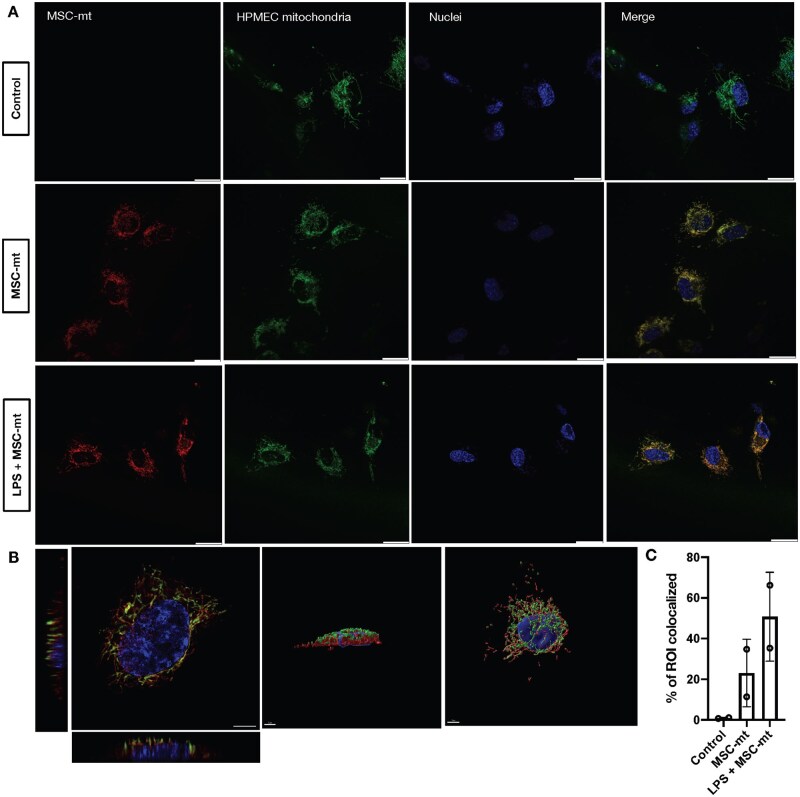
Mitochondria are efficiently internalized by HPMEC. (A) Representative live imaging of mitochondrial transplantation. Mitochondria isolated from MSC (MSC-mt) were prestained with MitoTracker Red and co-cultured with HPMEC prestained with MitoTracker Green for 24 h. Top panels: control. Middle panels: MSC-mt. Confocal imaging of internalized MSC-mt mitochondria (Mitotracker Red) co-localized to endogenous mitochondria network of non-stimulated HPMEC (MitoTracker Green). Lower panel: LPS + MSC-mt. MSC-mt internalized and co-localized to the endogenous mitochondrial network of HPMEC stimulated with LPS. The images were taken using Leica SP8 confocal microscope (Scale bar = 20 μm). (B) Representative images of orthogonal views (Scale bar = 20 μm) and 3D reconstruction (Scale bar = 5 μm) on the LPS + MSC-mt group. (C) Analysis of region of interest (ROI) colocalization of control, positive control, and MSC-mt group. Data presented as mean ± SD.

Next, we assessed the effects of MSC-mt transplantation on HPMEC in the presence of LPS. HPMEC were pre-stained with Mitotracker Green to visualize endogenous mitochondria, stimulated with LPS, and co-cultured with MSC-mt (pre-stained with Mitotracker Red) (Figure 2A). After 24 h, HPMEC readily internalized the exogenous MSC-mt, with a more pronounced effect in the presence of LPS (Figure 2A). Z-stacking plane demonstrated that MSC-mt were closely aligned with HPMEC mitochondrial network (Figure 2B), with at least 50.8% ± 21.9% of ROI colocalization between red and green Mitotracker fluorescence (Figure 2C). This data was supported by three-dimensional reconstruction and deconvolution (Figure S1, see online supplementary material for a color version of this figure), suggesting fusion between transplanted and endogenous mitochondria.

### Mitochondrial transplantation alleviates mitochondrial dysfunction in LPS-stimulated HPMECs without eliciting cytotoxicity or inflammatory response

**Figure 3.**
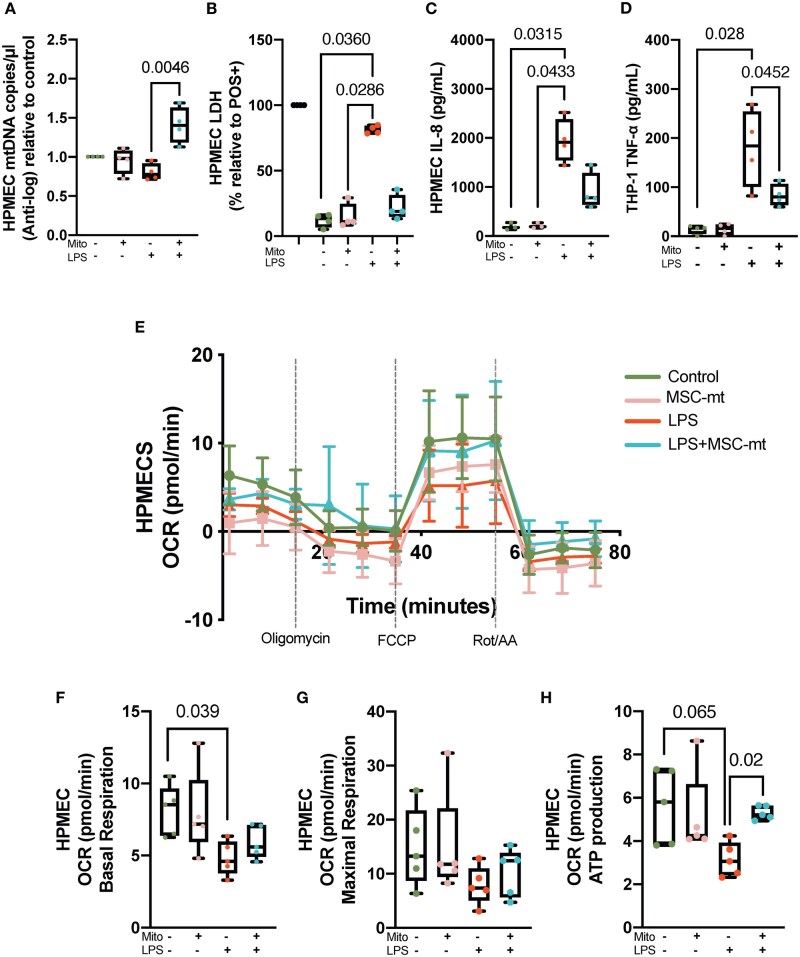
MSC-mt transplantation effects on mtDNA copies, viability, inflammatory activation, and mitochondrial respiration of recipient cells. (A) Quantification of mtDNA copy numbers in LPS-stimulated HPMEC after mitochondrial transplantation (24 h). (B) Effects on HPMEC viability measured by levels of LDH release. (*n* = 3–4). (C) Levels of interleukin (IL)-8 secretion by HPMEC measured by ELISA (*n* = 3–4). (D) Levels of TNF-α secreted by THP1 macrophages measured by ELISA (*n* = 3–4). (E) Representation of Seahorse Mito Stress assay curve showing OCR in HPMEC. (F–H) Values for respiratory parameters: basal respiration (F), maximal respiration (G), and ATP production (H) (*n* = 4–5). Data are illustrated as boxplots. The band indicates the median, the box indicates the interquartile range (IQR) of 25%–75%, and the whiskers denote the rest of the data distribution. Differences were assessed using Kruskal–Wallis with post-hoc Dunn’s test.

Co-culture with MSC-mt numerically increased mtDNA copy numbers in HPMEC stimulated with LPS (Figure 3A). Furthermore, mitochondrial transplantation did not elicit cytotoxicity, measured by the release of lactate dehydrogenase (LDH); however, it significantly reduced LDH release induced by LPS (Figure 3B).

Also, MSC-mt transplantation did not induce the release of pro-inflammatory cytokine IL-8 from HPMEC, but was able to markedly reduce IL-8 release upon LPS stimulation (Figure 3C). Similarly, MSC-mt did not induce inflammatory activation of the THP-1 cells (human monocyte cell line) measured by secretion levels of TNF-α cytokine, while significantly reducing its secretion in the presence of LPS, indicating an anti-inflammatory effect (Figure 3D).

Assessment of HPMEC mitochondrial respiration (Figure 3E) demonstrated that LPS significantly impaired basal (Figure 3F) and maximal oxygen consumption rates (Figure 3G), also reducing ATP turnover (Figure 3H). MSC-mt transplantation restored ATP turnover affected by LPS (Figure 3H).

**Figure 4.**
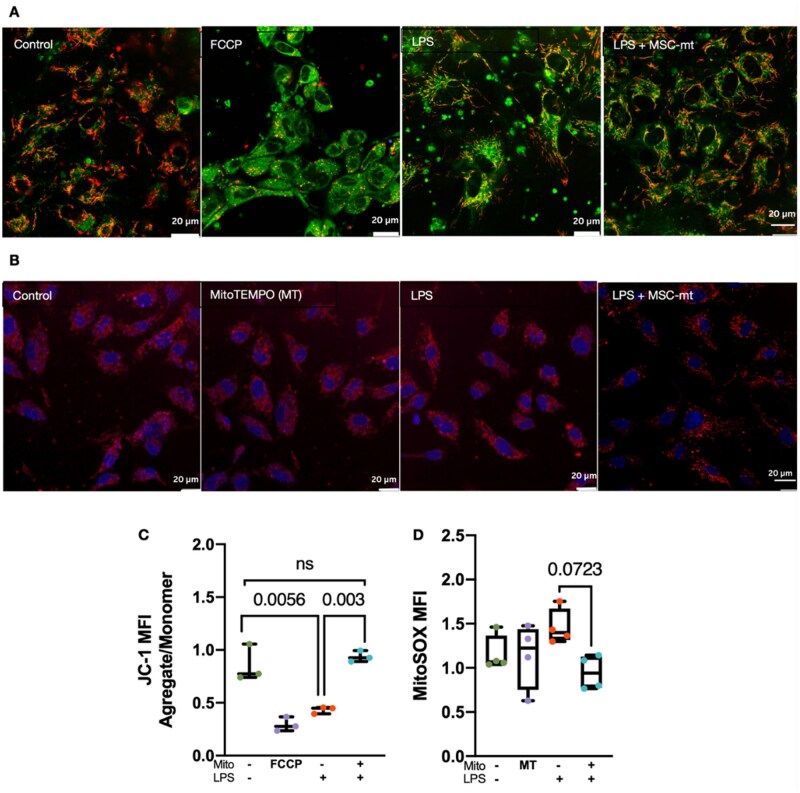
Mitochondrial transplantation alleviates mitochondrial dysfunction in HPMEC. (A) Representative images of JC-1 fluorescence in HPMEC. Green areas indicate depolarized mitochondrial membranes (JC-1 monomers), and red areas indicate polarized mitochondrial membranes (JC-1 aggregates). FCCP was used to induce mitochondria depolarization. Images taken using Leica SP8 confocal microscope (Scale bar = 20 μm). (B) Representative images of HPMEC mitochondrial superoxide production detected with MitoSOX. Mitotempo (MT) was used as positive control for ROS quenching. Images taken using Leica SP8 confocal microscope (Scale bar = 20 μm). (C) Quantification of red to green JC-1 fluorescence intensity ratio in HPMEC analyzed by ImageJ software. (D) Quantitative MitoSOX fluorescence intensity (MFI) analyzed by ImageJ software. Data are illustrated as boxplots. The band indicates the median, the box indicates the interquartile range (IQR) of 25%–75%, and the whiskers denote the rest of the data distribution. Differences were assessed using one-way ANOVA analysis with post hoc Bonferroni’s test.

Next, we investigated the effect of MSC-mt transplantation on HPMEC mitochondrial membrane potential. MSC-mt restored mitochondrial membrane potential affected by LPS (Figure 4A). Exposure of HPMECs to LPS also increased levels of mitochondrial ROS. However, although numerically lower, MSC-mt did not result in a significant reduction of ROS levels (Figure 4B). This data suggests that the proposed treatment may activate mechanisms beyond traditional antioxidant pathways.

### Mitochondria transplantation restores barrier integrity disrupted by LPS and ARDS plasma samples regardless of inflammatory subphenotype

**Figure 5.**
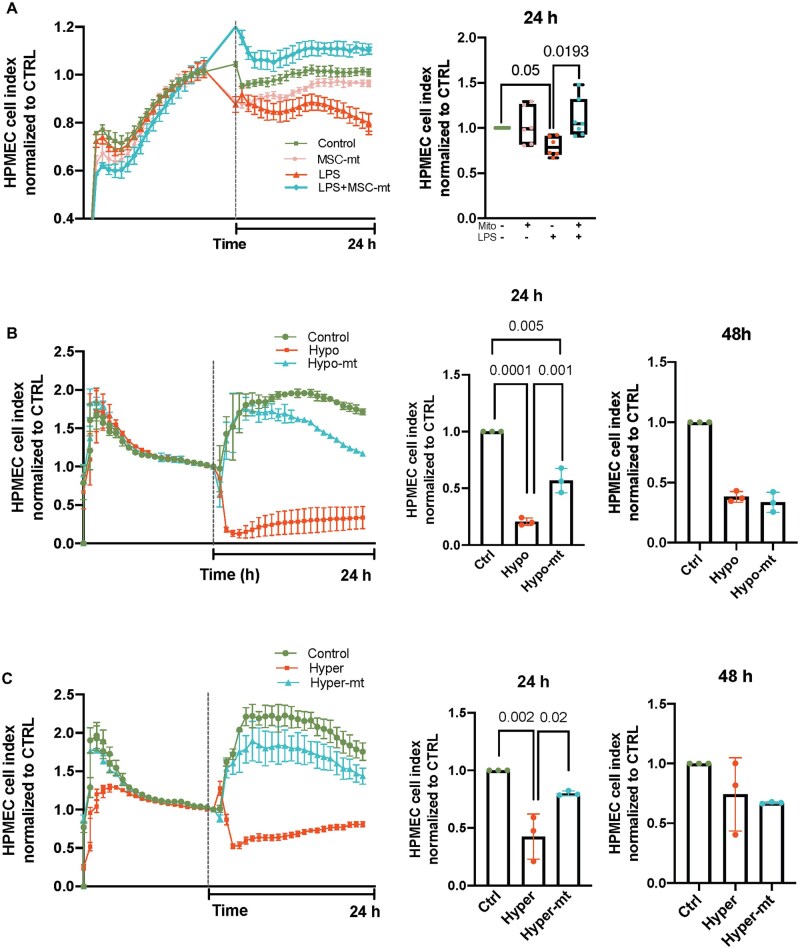
Mitochondrial transplantation restores HPMEC barrier integrity in the presence of LPS or ARDS plasma. (A) Real-time impedance analysis of HPMEC exposed to LPS and treated with MSC-mt and their respective cell impedance analysis of XCelligence RTCA measurements at a 24-h timepoint. Data are illustrated as boxplots. The band indicates the median, the box indicates the interquartile range (IQR) of 25%–75%, and the whiskers denote the rest of the data distribution. (B) Representative real-time impedance analysis of HPMEC exposed to hypoinflammatory ARDS plasma and their respective cell impedance analysis of XCelligence RTCA measurements at a 24 and 48-h timepoint. (C) Representative real-time impedance analysis of HPMEC exposed to hyperinflammatory ARDS plasma and their respective cell impedance analysis of XCelligence RTCA measurements at a 24 and 48-h timepoint. Data presented as mean ± SD. Differences were assessed using Kruskal–Wallis with post-hoc Dunn’s test (A) and one-way ANOVA analysis with post hoc Bonferroni’s test (B, C).

The main feature of pulmonary endothelial dysfunction in ARDS is increased permeability, which can result in vascular leakage and development of edema. LPS stimulation induced significant disruption of the barrier integrity of HPMECs, which was restored by MSC-mt transplantation (Figure 5A). To mimic human ARDS environment more closely, HPMEC were cultured for 24 and 48 h with 10% plasma from ARDS patients, previously categorized into hypo- or hyper-inflammatory phenotypes,^23^ while plasma from healthy volunteers served as a control. Exposure to both types of plasma samples induced comparable disruption of the barrier integrity in HPMECs, and mitochondria transplantation was able to restore the barrier integrity of endothelial cells at a 24-hour time-point. However, this effect was lost after 48 h from mitochondrial transplantation (Figure 5B and C).

### Mitochondria transplantation mitigates lung damage in a mouse model of injury induced by LPS

**Figure 6.**
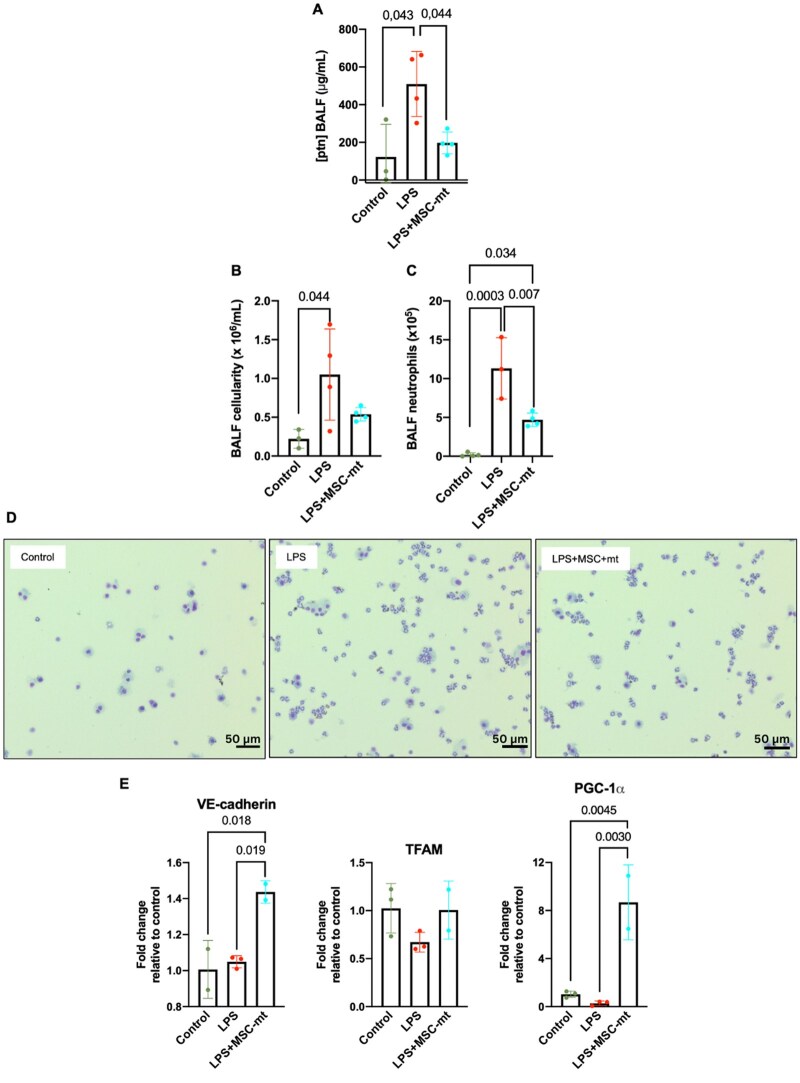
Mitochondrial transplantation mitigates lung damage in a mouse model of LPS-induced lung injury. (A) Total protein concentrations in the bronchoalveolar lavage fluid (BALF) samples. (B) Total cell counts and (C) absolute neutrophil counts in the BALF samples. (D) Representative images of BALF cytospin preparations. (E) mRNA expression of VE-cadherin, TFAM, and PGC-1α in mouse lung tissue (*n* = 3–4 mice per group). Data presented as mean ± SD. Differences were assessed using one-way ANOVA analysis with post hoc Bonferroni’s test.

To confirm if this beneficial effect of mitochondria transplantation will be relevant in vivo, we next used mouse LPS-induced acute lung injury model. To induce lung injury, C57BL/6 mice received intratracheal instillation of LPS and were treated with PBS or MSC mt intravenously (5 µg/g of BW) 4 h later. MSC-mt administration resulted in a significant reduction of LPS-induced lung injury and inflammatory cell infiltration into alveolar spaces, as evidenced by protein levels in the BALF (Figure 6A), as well as total inflammatory cell infiltration and absolute neutrophil counts (Figure 6B and C). Cytospin analysis of the BALF revealed substantial recruitment of inflammatory cells to the alveolar compartment, predominantly neutrophils in the LPS-injured group, which was attenuated by administration of MSC-mt (Figure 6D).

Interestingly, MSC-mt transplantation resulted in enhanced mRNA expression of VE-cadherin (as an indirect measure of endothelial functionality) in the lung tissue as well as increased expression of *TFAM* and *PGC-1a mRNA* (genes regulating mitochondrial biogenesis) (Figure 6E). Together, these data indicate pulmonary endothelial barrier recovery after mitochondria transplantation, associated with recovery of mitochondrial biogenesis at the lung tissue level.

## Discussion

The main findings of this study are the following: (1) mitochondria were successfully isolated from MSC, maintaining healthy membrane potential and structure; (2) mitochondria were rapidly internalized by HPMECs after 24 h of co-culture and integrated into the endogenous mitochondria network; (3) MSC-mt transplantation alleviated inflammatory response, and mitochondrial dysfunction, and recovered barrier integrity of HPMECs in the presence of LPS; (4) stimulation with plasma from ARDS patients disrupted the barrier of HPMEC, and mitochondria transplantation was able to restore barrier integrity in the presence of both plasma subphenotypes (hyper and hypo-inflammatory) over 24 h; and (5) mitochondrial transplantation was also beneficial in the in vivo induced model of ARDS, reducing neutrophil infiltration and BALF cellularity content, added to a lung tissue modulation of VE-cadherin, and mitochondrial biogenesis genes *TFAM* and *PGC-1α*, suggesting restoration of the pulmonary endothelial barrier at tissue level, which can be partially explained by recovery of mitochondrial biogenesis pathways. In the present study, transplantation of healthy mitochondria demonstrated therapeutic potential for pulmonary endothelial dysfunction in ARDS.

Mitochondrial transplantation approach has been intensively discussed in the past few years, especially after the pilot clinical study showing its therapeutic effects in pediatric patients with cardiogenic shock.^25^^,^^26^ Although extracellular mitochondria and their derived products have been recognized as pro-inflammatory signals related to damage-associated molecular patterns (DAMPs), which makes the concept of mitochondrial transplantation counterintuitive,^27^ accumulating evidence demonstrates therapeutic effects (including anti-inflammatory effects) of mitochondrial transplantation in different systems.^28–30^ Our data showed that mitochondrial transplantation attenuated LPS-induced inflammation in HPMEC and THP1-derived macrophages. This is an important highlight, once high mortality in acute lung injury results from sustained pro-inflammatory signaling, especially by the cytokines IL-8 and TNF-α.^31^ IL-8 is a potent neutrophil chemotactic factor and a consistent and reliable biomarker of ARDS severity,^32^^,^^33^ with its concentrations significantly associated with mortality in sepsis and pneumonia.^34^ Likewise, TNF-α levels are increased in ARDS patients and are involved in the reduction of barrier function through the induction of instability of cell junction proteins.^35^ Recent studies have demonstrated that mitochondrial transplantation successfully reduces mRNA expression and secretion of pro-inflammatory cytokines in alveolar macrophages by modulating macrophage polarization in an in vitro model of ARDS. These results are consistent with our findings, demonstrating a similar impact of mitochondrial transplantation on controlling active inflammatory cells in vitro and in vivo.^36^ Therefore, mitochondrial transplantation showed potential in mitigation of the inflammatory response present in ARDS.

In the present study, mitochondrial transplantation in HPMEC primed with LPS increased total mtDNA content, improved mitochondrial health, and attenuated metabolic dysfunction related to ATP production. These results are consistent with Caicedo et al.^22^ showing that after transplantation of mitochondria isolated from MSC, MDA-MB-231 cancer cells increased total mtDNA content. The study concluded that the observed increase in the total mtDNA was a result of augmentation in endogenous MDA-MB-231 mtDNA, suggesting transplantation of exogenous mitochondria facilitated replication of the endogenous mtDNA, possible through modulation of mitochondrial biogenesis or inhibition of mtDNA degradation.

Endothelial dysfunction and hyperpermeability are some of the main hallmarks of ARDS. Interestingly, Hough et al.^6^ showed mitochondrial dysfunction as a main contributor to the failure of the endothelial barrier in the context of acute lung injury and inducing hyperpermeability. Therefore, the maintenance of mitochondrial health is intrinsically associated with the physical function of the endothelial barrier, making it a promising target for novel therapies. Our study showed the transient beneficial effects of mitochondrial transplantation on the barrier integrity of HPMEC disrupted with ARDS plasma after 24 h, suggesting that a single administration may not be sufficient for a lasting therapeutic effect. However, these data also suggest that both subphenotypes of ARDS patients would be equally responsive to Mt therapy. Consistent with our in vitro data, mitochondria transplantation significantly alleviates lung injury in vivo resulting in a significant reduction of BALF protein concentrations and alveolar inflammatory cell infiltration. These data are in line with previous evidence demonstrating the therapeutic effects of mitochondrial transfer from MSC or MSC-EVs in in vivo models of ALI/ARDS.^8^^,^^12^^,^^16^^,^^36^ The study by de Carvalho et al.^18^ also reported similar therapeutic effects of mitochondria-enriched fraction isolated from murine MSCHP using a model of cecal ligation and puncture (CLP) to induce sepsis. Most recently, Lee et al.^36^ have reported protective effects of Mt isolated from umbilical cord MSC and induced pluripotent cells in a similar model of LPS-induced lung injury in mice. In this study, mitochondrial transplantation did not reduce inflammatory cell infiltration into the airspaces, interestingly authors detected a significant reduction of TNF-α and IL-6 levels in the BALF with Mt isolated from iPSCs but not from MSC. Together, there is accumulating evidence showing the potential of mitochondrial transplantation in vivo in different models of ARDS.

Interestingly, we found an increase in the expression of VE-cadherin in the lung tissue induced by mitochondrial transplantation, suggesting recovery of the endothelial barrier at the tissue level. It was accompanied by an increase in TFAM and PGC-1α expression, indicating that stimulation of mitochondrial biogenesis pathways may be partially related to the positive effects of mitochondrial transplantation on the endothelial barrier integrity. These results are also consistent with previous in vitro studies showing that mitochondrial transplantation seems to initiate mitochondrial biogenesis pathways.^22^ However, an in-depth investigation of the molecular pathways involved in mitochondrial transplantation is still needed to better understand its therapeutic benefits.

Our study has limitations. The molecular mechanisms involved in mitochondrial uptake and mechanisms of action were not investigated in this study. The effects of isolated MSC mitochondria were not compared to mitochondria isolated from other cell sources; however, preliminary study (data not shown) demonstrated suboptimal and non-consistent effects of mitochondria isolated from fibroblasts compared to MSC mitochondria (data not shown). LPS model of lung injury does not reflect the complexity of human ARDS, and it was used as a proof of concept to test the potential of mt transplantation.

## Conclusions

MSC-mitochondria transplantation alleviated mitochondrial dysfunction, and inflammatory activation, improved survival and restored barrier properties of primary human pulmonary endothelial cells in the inflammatory environment in vitro, and alleviated severity and inflammatory response in the model of lung injury in vivo. These results suggest the possibility of MSC mitochondria becoming a novel MSC-based therapy for inflammatory conditions. Further studies are needed to evaluate mitochondrial transplantation as a potential therapeutic strategy for ARDS.

## Supplementary Material

szaf053_Supplementary_Data

## Data Availability

All data generated in this study are included in the article and its supplementary data files. All raw data are available upon reasonable request.
