# Supplementary material for: Transplantation of mesenchymal stromal cell-derived mitochondria alleviates endothelial dysfunction in pre-clinical models of acute respiratory distress syndrome
Source: Stem Cells Transl Med. 2025 Nov 15;14(11):szaf053. doi: 10.1093/stcltm/szaf053 (PMC12618169; doi:10.1093/stcltm/szaf053)
Supplement: szaf053_Supplementary_Data [file szaf053_supplementary_data.pdf]

TRANSPLANTATION OF MSC MITOCHONDRIA ALLEVIATES ENDOTHELIAL DYSFUNCTION IN PRE-CLINICAL MODELS OF ACUTE RESPIRATORY DISTRESS SYNDROME

Dayene de Assis Fernandes Caldeira, *PhD* [1,2], Johnatas Dutra Silva, *PhD* [1,2], Monique Martins Melo, *PhD*[2,3], Rodrigo Gonzaga Veras [2,3], Daniel F. McAuley, *MD* [1], Patricia Rieken Macedo Rocco, *PhD* [2,3], Pedro Leme Silva, *PhD* [2,3], Fernanda Ferreira Cruz, *PhD* [2,3#], Anna Krasnodembskaya, *PhD* [1#]

[1]*Wellcome-Wolfson Institute for Experimental Medicine, School of Medicine, Dentistry, and Biomedical Sciences, Queen's University Belfast, Belfast, UK*

[2]*Laboratory of Pulmonary Investigation, Institute of Biophysics Carlos Chagas Filho, Federal University of Rio de Janeiro, Rio de Janeiro, Brazil*

[3]*National Institute of Scien*ce and Technology for Regenerative Medicine, Rio de Janeiro, Rio de Janeiro, Brazil

[#]Corresponding authors:

Fernanda Ferreira Cruz: *ffcruz@biof.ufrj.br*
Anna Dmitrievna Krasnodembskaya: *a.krasnodembskaya@qub.ac.uk*

Running Title: MSC mitochondria mitigate endothelial injury in ARDS

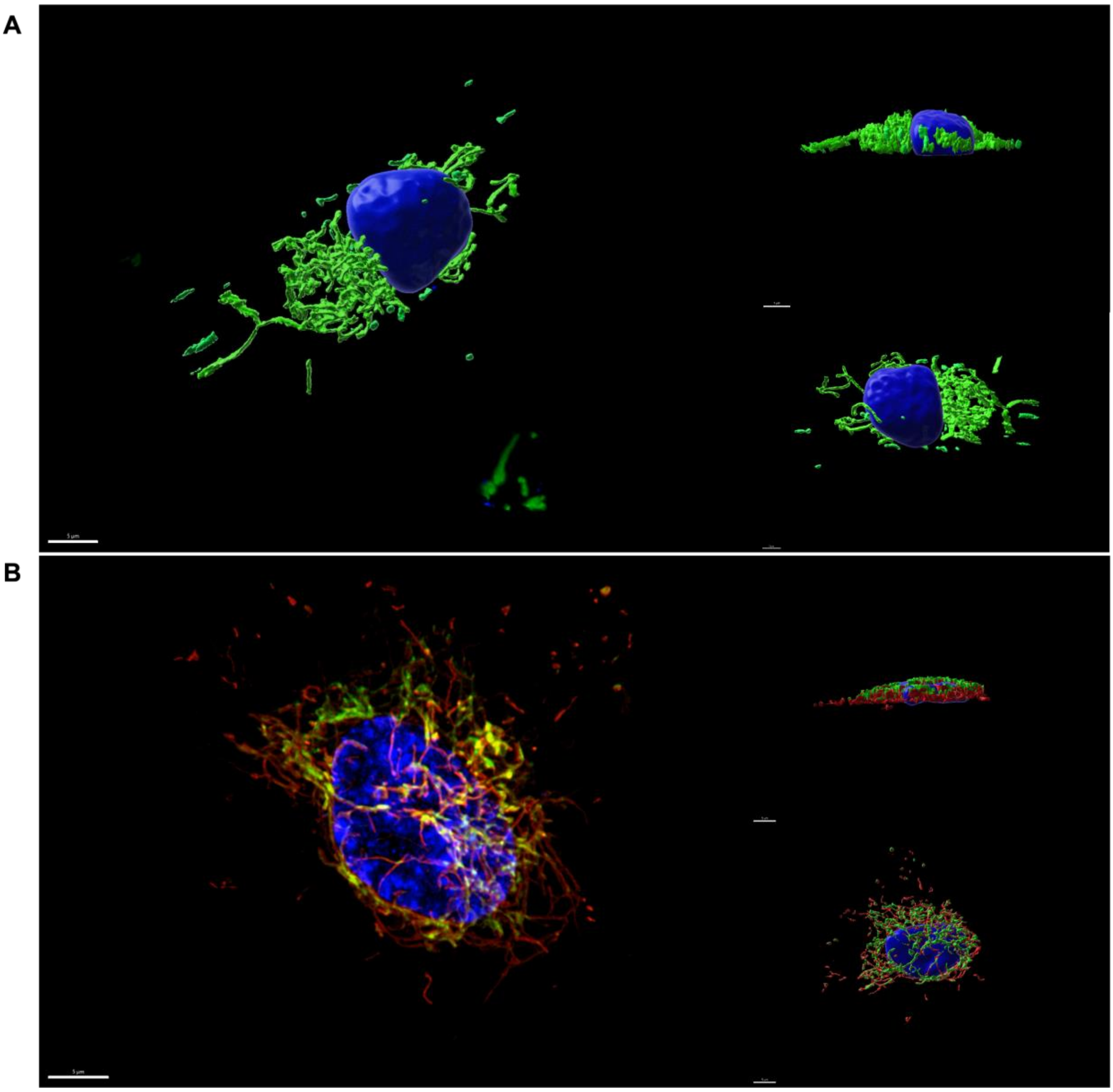

S1. Representative 3D reconstruction (iMARIS) live imaging. (a) HPMEC control and (b) after LPS exposure and mitochondrial transplantation in different focal planes by z-stack technique (Scale bar = 5 µm, 100x). HPMECs exposed to LPS and treated with mitochondrial transplantation showed after 24 hours exogenous mitochondria derived from hBMSCs (red) colocalized with HPMEC endogenous mitochondria (green).
